# Methods of muscle spasticity assessment in children with cerebral palsy: a scoping review

**DOI:** 10.1186/s13018-024-04894-7

**Published:** 2024-07-11

**Authors:** Mehdi Nourizadeh, Babak Shadgan, Samin Abbasidezfouli, Maria Juricic, Kishore Mulpuri

**Affiliations:** 1 Implantable Biosensing Laboratory, ICORD, Vancouver, Canada; 2 Department of Pathology and Laboratory Medicine, University of British Columbia, Vancouver, Canada; 3 The Heart and Lung Innovation Centre, Department of Anesthesiology, Pharmacology, and Therapeutics, University of British Columbia, Vancouver, Canada; 4 Department of Orthopaedics, University of British Columbia, Vancouver, Canada; 5 Department of Physical Therapy, University of British Columbia, Vancouver, Canada; 6 Department of Orthopaedic Surgery, BC Children’s Hospital, University of British Columbia, Vancouver, Canada

**Keywords:** Cerebral palsy, Muscle spasticity, Objective techniques, Subjective methods

## Abstract

**Background:**

Evaluating muscle spasticity in children with cerebral palsy (CP) is essential for determining the most effective treatment strategies. This scoping review assesses the current methods used to evaluate muscle spasticity, highlighting both traditional and innovative technologies, and their respective advantages and limitations.

**Methods:**

A search (to April 2024) used keywords such as muscle spasticity, cerebral palsy, and assessment methods. Selection criteria included articles involving CP children, assessing spasticity objectively/subjectively, comparing methods, or evaluating method effectiveness.

**Results:**

From an initial pool of 1971 articles, 30 met our inclusion criteria. These studies collectively appraised a variety of techniques ranging from well-established clinical scales like the modified Ashworth Scale and Tardieu Scale, to cutting-edge technologies such as real-time sonoelastography and inertial sensors. Notably, innovative methods such as the dynamic evaluation of range of motion scale and the stiffness tool were highlighted for their potential to provide more nuanced and precise assessments of spasticity. The review unveiled a critical insight: while traditional methods are convenient and widely used, they often fall short in reliability and objectivity.

**Conclusion:**

The review discussed the strengths and limitations of each method and concluded that more reliable methods are needed to measure the level of muscle spasticity more accurately.

**Supplementary Information:**

The online version contains supplementary material available at 10.1186/s13018-024-04894-7.

## Introduction

Cerebral palsy (CP) is a group of permanent, nonprogressive neurological disorders that affect movement, posture, and muscle coordination and limit the activities of daily living, leading to dysmorphisms [1–3]. The incidence rate of CP is 2–2.5% per 1000, making it the most common disorder leading to physical disability in children [1–5]. It is caused by damage or abnormalities in the developing brain, usually occurring before or during birth, but can also occur during early childhood. The condition is characterized by varying degrees of motor impairment, which can range from mild to severe. Specifically, damage to the motor cortex, which is responsible for planning, executing, and controlling voluntary movements, can lead to muscle spasticity in CP. Spasticity is a condition with a constant state of muscle contraction, resulting in stiffness and difficulty in movement [6]. The motor cortex is located in the brain's frontal lobe, and damage to this area can result in abnormalities in muscle tone, posture, and other motor-related symptoms. However, it is worth noting that CP can result from damage to different parts of the brain, and the specific location of the brain injury can affect the severity and type of motor symptoms that develop, including muscle spasticity [1, 7].

Complications of CP include communication difficulties, gastrointestinal abnormalities, and bone conditions such as osteopenia [1, 8]. Individuals with CP often experience difficulties with muscle tone, control, balance, and coordination, leading to challenges in walking, speaking, eating, and performing everyday activities. The specific symptoms and severity of CP can vary from person to person, as the location and extent of the brain damage determine the areas of the body affected. Muscle spasticity in children with CP can also cause a wide variety of discomforts ranging from pain to hip displacement, which requires medical intervention to improve the dynamics and quality of life of patients [5, 7, 8].

Among different treatment options, such as physical therapy, medication, the Bobath neurodevelopmental method, and surgical procedures used to manage spasticity, the appropriate remedy is selected according to each individual’s specific symptoms [1, 8]. Botulinum toxin type A and baclofen are the medications commonly administered for managing spasticity in children with CP [1, 3]. Botulinum toxin (Botox), a formulation of botulinum toxin type A from the bacterium Clostridium botulinum, can prevent acetylcholine release from nerve terminals and relax muscles [3, 4]. Although Botulinum neurotoxin type A (BoNT A) is not an FDA-approved treatment option for children with CP, it is still considered one of the best options due to its long-lasting effect, noninvasiveness, cost-effectiveness, and accessibility [3]. For over 2 decades, BoNT A has been used to treat spasticity in individuals with CP younger than 19 years [9, 10]. Different clinicians use varying dosages, measured in units of activity and injected volumes, to treat muscle spasticity based on their evaluation of spasticity [10].

Developing a spasticity management plan and finding the optimal procedure for each individual is highly dependent on the level of spasticity. A central challenge, in assessing muscle spasticity in children with CP, is the lack of a universally accepted definition of spasticity, an issue highlighted in an interdisciplinary workshop held at the National Institutes of Health in April 2001 [11]. Current methods for scaling the level of spasticity, such as the modified Ashworth Scale and the modified Tardieu Scale, add some insight but do not provide accurate information physicians need to define the dosage and timing of therapeutic interventions. In recent years, several quantitative scales such as pendulum test, Australian Spasticity Assessment Scale and real-time sonoelastography have been proposed for objective assessment and scaling of spasticity but have yet to gain widespread use in clinical applications.

The works of Scholtes et al. [12], and Aloraini et al. [13]. collectively underline the complexity and diversity in spasticity measurement methods. While these studies emphasize the critical role of precise and comprehensive assessment tools, they also reveal significant gaps in the development and validation of these tools, particularly concerning their reliability and validity across varied clinical settings. Our scoping review was conceived in response to these gaps, aiming to provide an updated and thorough overview of the latest methodologies and technologies in the field. We specifically targeted the integration of novel, objective measures and the standardization of assessment protocols. Our study not only enriches the existing body of knowledge by cataloging and critiquing current methodologies but also pioneers in identifying and recommending future directions for research.

This scoping review aims to map the available subjective scales and objective measures for assessing spasticity in children with CP.

## Method

This scoping review was conducted according to the framework outlined by Arksey and O’Malley [14] and extended by Levac et al. [15]. In addition, this review is reported according to the PRISMA extension for a scoping review [16]. We did not develop a protocol for this scoping review.

### Research question

The primary research question for this article review was: “What are the most effective methods for assessing muscle spasticity in children with CP?”.

To further clarify and support this primary question, we identified two sub-questions that guide the scope of our review. These questions were:

1. How do various subjective and objective methods compare in accuracy and reliability for assessing muscle spasticity in children with CP?
2. What are the strengths and limitations of current spasticity assessment tools in clinical and research settings?

### Inclusion criteria

#### Participants

Studies involving children and adolescents (0–18 years of age) with CP. This includes all types and severities of CP.

#### Concept

The focus of studies must be on assessing muscle spasticity using either objective or subjective measures. Studies should either compare different assessment methods or evaluate the effectiveness, reliability, or validity of a particular assessment method.

#### Context

Included studies conducted in any clinical or research setting, including hospitals, rehabilitation centers, outpatient clinics, and research laboratories. The review is interested in studies conducted in diverse geographical locations and healthcare settings to understand the global applicability of the assessment methods.

#### Type of sources

The review includes original research articles, systematic reviews, meta-analyses, cohorts, and clinical trials. Case studies, editorials, commentaries, and letters are excluded. The review considers studies published in English, given the language capabilities of the research team.

### Search strategy

We searched PubMed, Web of Science Core Collection and Google Scholar. The search strategy did not place limitations. The search included a combination of keywords related to muscle spasticity, CP, and assessment methods the date of the last search was April 2024. See Supplement 1 for the search strategy used in databases. To ensure that we located all relevant sources of evidence, we consulted Dr. Shadgan and Dr. Mulpuri who are experts on our team for suggestions of papers that may have been missed by our search, and we performed hand-searching of reference lists of relevant articles to identify studies related to our objective.

### Study selection

To determine eligibility, we used a two-step process. First, we assessed the titles and abstracts followed by the full text against the inclusion criteria. At each stage, each reference was screened by two members independently and in duplicate. To remove duplicates, references were imported to Covidence, duplicate entries were automatically detected and highlighted. To ensure accuracy, a manual review has been done, following which duplicates were excluded. Any disagreements were resolved through consultation with a third senior investigator.

### Data extraction

After identifying the final articles, two independent investigators carefully examined each article's findings, methods, and the specific assessment scales they utilized. The key findings of the articles, participants, and the method of spasticity assessment were extracted, and summaries of information extracted from studies were provided. Any disagreements were resolved through consultation with a third senior investigator.

### Evaluation of the levels of evidence?

The Oxford Centre for Evidence-Based Medicine (OCEBM) criteria were also used to critically evaluate the levels of evidence of the included research [17]. The OCEBM guidelines are divided into five levels, from Level 1 (highest) to Level 5, each corresponding to a particular study design [18]. For example, case studies or expert opinions are located at the bottom of the hierarchy (Level 5), whereas randomized controlled trials (RCTs) are found at the top (Level 1). Table 1 illustrates various levels of OCEBM.

**Table 1:** OCEBM levels of evidence. *Source*: Adapted from OCEBM levels of the Evidence Working Group [18]

Level	Type of study
1a	SR/MA of RCTs
1b	Individual RCT
2a	SR/MA of cohort studies
2b	Individual cohort study (including low quality RCT)
3a	SR/MA of case–control studies
3b	Individual case–control study
4	Case series (and poor-quality cohort and case–control studies)
5	Expert opinion without explicit critical appraisal, or based on physiology, bench research or ‘first principals’

*OCEBM* Oxford Centre for Evidence-Based Medicine, *MA* meta-analysis, *RCT* randomized controlled trial, *SR* systematic review

Two independent reviewers from our research team were involved in this evaluation process. Each study was individually assessed by these reviewers, who then compared their evaluations to ensure consistency and objectivity. The involvement of two reviewers aimed to minimize bias and enhance the reliability of our evidence grading.

In cases where the two reviewers had differing opinions on a study's level of evidence, a structured discussion was held to reach a consensus. If a consensus could not be achieved through discussion, a third senior investigator was consulted to provide an additional perspective and facilitate a resolution.

## Results

### Study selection

**Fig. 1:**
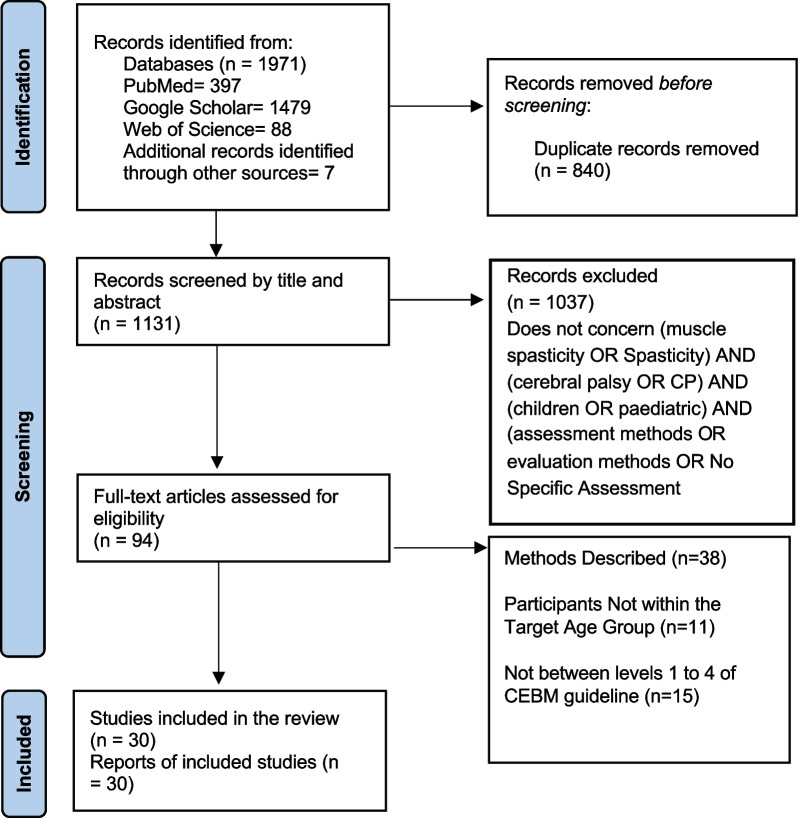
PRISMA 2020 flow diagram—methodology used for the selection and inclusion of articles. Databases include PubMed, Web of Science and Google Scholar/CP: Cerebral Palsy/N: Number of papers reviewed by the authors at each step

Initially, 1971 primary titles were identified, which were narrowed down to 94 papers related to CP and assessments of muscle spasticity. Additional articles were found by reviewing references. Ultimately, 30 articles were deemed relevant to evaluating muscle spasticity in children with CP and were used as the basis for the review. Figure 1 describes the methodology used for the selection and inclusion of articles.

### Study characteristics and quality assessment

The 30 included studies and their characteristics are summarized in Table 2. All the articles included in the qualitative analysis were published from 1999 to April 2024. All studies included children with CP, both male and female, with ages ranging from 1 to 19 years old. The mean sample size was 29.86, ranging from 10 to 168. Various CP subtypes were explored, prominently focusing on spastic hemiplegia and spastic diplegia, and the studies involved a range of clinical and instrumented techniques to assess qualitative and quantitative aspects.

**Table 2:** Summary of study characteristics and their Oxford Centre for Evidence-Based Medicine (OCEBM) scores

Article	Study	Participants	Key findings	OCEBM score
Graham et al. [5]	Does botulinum toxin A combined with bracing prevent hip displacement in children with cerebral palsy and “hips at risk”? A randomized, controlled trial	91 children with CP Male = 59 Female = 32 All spastic cp; 29 children with diplegia and 62 children with quadriplegia	Intramuscular botulinum toxin A injections combined with comprehensive rehabilitation therapy improved gait patterns and functional outcomes in children with cerebral palsy and spastic diplegia using modified Ashworth scale and Modified Tardieu scale	1b
Bjornson et al. [8]	Botulinum toxin for spasticity in children with cerebral palsy: a comprehensive evaluation	33 children with spastic CP; diplegia Male = 18 Female = 15	BTX-A treatment resulted in significant decreases in spasticity using the Modified Ashworth scale and PROM, improvement in motor function and strength, and changes in functional ability, but these changes did not consistently translate into increased satisfaction with treatment outcomes among the patients and their families	2b
Szopa et al. [19]	Quadriceps femoris spasticity in children with cerebral palsy: measurement with the pendulum test and relationship with gait abnormalities	36 children with CP including 18 children with spastic hemiplegia and 18 spastic diplegia Male = 22 Female = 14	Accelerometer-based pendulum test can effectively differentiate between normal knee extensor muscles and those with spasticity in children with cerebral palsy and can also distinguish between various degrees of spasticity, such as spastic hemiplegia and spastic diplegia, but there is no correlation between the degree of knee extensor spasticity and deviations from normal gait in children with cerebral palsy	2b
Bilgici et al. [20]	Quantitative assessment of muscle stiffness with acoustic radiation force impulse elastography	12 children with spastic CP; diplegic Male = 6 Female = 6	ARFI elastography for assessing spasticity can yield more valuable information with the combined use of MAS	4
Pandyan et al. [21]	A review of the properties and limitations of the Ashworth and modified Ashworth Scales as measures of spasticity	NA	While the Ashworth Scales can effectively measure resistance to passive movement, they do not provide an exclusive measurement of spasticity	1a
Gracies et al. [22]	Reliability of the Tardieu Scale for assessing spasticity	Pretraining phase: 5 children with spastic CP Male = 3 Female = 2 Training phase: 3 children with spastic CP Post-training phase: 15 children with spastic CP Male = 7 Female = 8	Both parameters of the Tardieu Scale had excellent intrarater and interrater reliability when assessed at the elbow and ankle joints of children with CP	2b
Tendroff et al. [23]	Synergistic muscle activation during maximum voluntary contractions in children with and without spastic cerebral palsy	31 participants 18 children with spastic CP including 12 with diplegia and 6 with hemiplegia Male = 12 Female = 6 13 without CP Male = 7 Female = 6	Children with spastic cerebral palsy demonstrate altered patterns of muscle U Activation and shortened latencies of synergistic muscle activation during maximum voluntary contractions, using EMG (electromyography), particularly affecting distal leg muscles, compared to typically developing children	2b
Rahimi et al. [24]	Objective assessment of spasticity by pendulum test: a systematic review on methods of implementation and outcome measures	NA	Pendulum test is a promising method for objectively assessing spasticity, but there is a lack of standardization in its implementation and outcome measures, and further research is needed to optimize its clinical use	2a
Park and Kwon [25]	Application of real-time sonoelastography in musculoskeletal diseases related to physical medicine and rehabilitation	NA	Real-time sonoelastography (RTS) is a promising imaging modality for evaluating tissue elasticity in various musculoskeletal diseases, providing valuable information for clinical assessment and potentially aiding in the diagnosis and monitoring of conditions such as myofascial pain syndrome, myopathy, spastic cerebral palsy, congenital muscular torticollis, lymphedema, and osteoarthritis	4
Kwon and Kwon [26]	Botulinum Toxin an Injection Combined with Radial Extracorporeal Shock Wave Therapy in Children with Spastic Cerebral Palsy: Shear Wave Sonoelastographic Findings in the Medial Gastrocnemius Muscle, Preliminary Study	15 children with spastic CP Male = 8 Female = 7	Combining botulinum toxin A (BTA) injection with extracorporeal shock wave therapy (ESWT) results in a more significant reduction in muscle spasticity and stiffness, as assessed by both the Modified Ashworth Scale (MAS) and Shear-Wave Sonoelastography (SWS), in children with spastic cerebral palsy compared to BTA injection alone	2b
Domagalska et al. [27]	relationship between clinical measurements and gait analysis data in children with cerebral palsy	36 children with CP as participants. Among them, 18 had spastic hemiplegia (HS) and 18 had spastic diplegia (DS) Male = 22 Female = 14	Gait pathology in children with cerebral palsy does not depend on the static and dynamic contractures of hip and knee flexors, and there is only a fair to moderate correlation between the spasticity of the rectus femoris muscle and deviations from normal gait patterns (they used DAROM)	2b
Alhusaini et al. [28]	Evaluation of spasticity using Ashworth and Tardieu scales compared with laboratory measures	27 independently ambulant children with spastic CP Male = 17 Female = 10	The Tardieu Scale was effective in identifying the presence of spasticity and contracture and the severity of contracture but was not able to identify the severity of spasticity	2b
Fosang et al. [29]	Measures of muscle and joint performance in the lower limb	18 children with spastic CP Male = 7 Female = 11	Interrater reliability for PROM and MTS techniques was acceptable	3b
Mutlu et al. [30]	Reliability of Ashworth and Modified Ashworth scales	38 children with spastic diplegic CP Male = 27 Female 11	Ashworth and modified Ashworth scales are not very reliable and assessments of spasticity using these scales should be interpreted with caution	4
Fowler et al. [31]	Sensitivity of the pendulum test for assessing	30 children with spastic CP including 23 with diplegia, 3 with hemiplegia, and 4 with quadriplegia, 10 children without CP Male and Female = NA	The first swing excursion in the Pendulum test was the best predictor of the degree of spasticity in persons with CP	2b
Park and Kwon [32]	Sonoelastographic evaluation of medial gastrocnemius muscles intrinsic stiffness	17 children with spastic CP; including 12 with diplegia and 5 with hemiplegia Male = 10 Female = 7	Muscle spasticity can be estimated using RTS combined with clinical scale measurements	2b
Jang et al. [33]	Usefulness of the Tendon Reflex for Assessing Spasticity After Botulinum Toxin-A Injection in Children with Cerebral Palsy	40 children with spastic CP aged 2–18 years Male = 26 Female = 14	Neurophysiologic assessments including Hoffmann and tendon reflex could be useful to assess spasticity after botulinum toxin-A injection	2b
Love et al. [34]	Interobserver reliability of the Australian Spasticity Assessment Scale (ASAS)	22 children with spastic CP Male = 16 Female = 6	Australian Spasticity Assessment Scale (ASAS) is a reliable and easy-to-use clinical tool for identifying and quantifying spasticity in children with cerebral palsy, demonstrating high interrater reliability among three experienced physiotherapists in assessing various muscle groups of the upper and lower limbs	4
Alorani et al. [13]	Spasticity Measurement Tools and Their Psychometric Properties Among Children and Adolescents With Cerebral Palsy: A Systematic Review	NA	The current state of spasticity assessment lacks a method that possesses the necessary psychometric properties and is easily used in clinical settings	2a
Drefus et al. [35]	The Root-Ely Modified Test of Rectus Femoris Spasticity Has Reliability in Individuals with Cerebral Palsy	20 children with Spastic CP Male = 12 Female = 8	Root-Ely scale has acceptable intra- and inter-rater reliability in pediatric individuals with cerebral palsy among experienced clinicians	4
Marsico et al. [36]	Hypertonia Assessment Tool: Reliability and Validity in Children With Neuromotor Disorders	46 participants with neuromotor disorders Male = 30 Female = 16	HAT showed moderate to substantial interrater reliability and almost perfect intrarater reliability. The validity of the HAT for detecting spasticity was confirmed, but for dystonia and rigidity, further studies were suggested	2b
Tsai et al. [37]	Clinical validity of a 0–10 numeric rating scale measure of spasticity in children with cerebral palsy	50 Children with CP Male = 28 Female = 22	The caretaker-reported proxy 0–10 NRS showed a significant correlation with MAS, while the child-reported 0–10 NRS showed a significant correlation with the Tardieu R2 (range of motion). Neither of the 0–10 NRS measurements showed significant correlations to spasticity as determined by the Tardieu Scale	2b
Schmartz et al. [38]	Measurement of muscle stiffness using robotic assisted gait orthosis in children with cerebral palsy: a proof of concept	10 children with spastic CP Male = 5 Famle = 5	High reliability for hip and knee movements using the assessment tool. A significant decrease in muscle stiffness, particularly in children with high levels of muscle tone, was observed after a single robotic-assisted gait training session	2b
Jobin and Levin [39]	Regulation of stretch reflex threshold in elbow flexors in children with cerebral palsy: a new measure of spasticity	14 children with spastic CP Male and Female = NA	Good test–retest reliability for the stretch reflex threshold measurements (ICC 0.73). However, a significant correlation between these measurements and clinical spasticity scales was not found	2b
van den Noort et al. [40]	Evaluation of clinical spasticity assessment in Cerebral palsy using inertial sensors	20 children with spastic CP Male and female = NA	Goniometry is an imprecise method to measure the true Angle of Catch in spasticity assessment, mainly due to joint repositioning after fast muscle stretch. The IS were suggested as a better alternative when a precise measurement of the AOC is required	2b
Lynn et al. [41]	Comprehensive quantification of the spastic catch in children with cerebral palsy	46 children with spastic CP Male = 22 Female = 24	Objective parameters can define and quantify the severity of the spastic catch in children with CP	2b
Cherni et al. [42]	Intra- and inter-tester reliability of spasticity assessment in standing position in children and adolescents with cerebral palsy using a pediatric exoskeleton	16 children with spastic CP Male = 9 Female = 7	L-STIFF tool showed moderate to excellent intra-tester reliability and fair to good inter-tester reliability. It was noted that the tool's reliability was better during fast and medium movement speeds compared to slow speeds	2b
Germanotta et al. [43]	Spasticity Measurement Based on Tonic Stretch Reflex Threshold in Children with Cerebral Palsy Using the PediAnklebot	10 children with spastic cerebral palsy Male and Female = NA	The study demonstrated the feasibility of using the PediAnklebot for assessing spasticity in plantar flexor muscles at the ankle joint in children with CP. No significant correlation was found between the objective measures and the MAS scores	2b
Wang et al. [44]	Acupuncture and Tuina Treatment for Gross Motor Function in Children with Spastic Cerebral Palsy: A Monocentric Clinical Study	168 children with spastic CP Male = 81 Female = 87	Treatment with acupuncture and tuina showed significant improvements in Modified Ashworth Scale, Gross Motor Function Measure D and E, six minute walking distance, and Modified Children’s Functional Independence Rating Scale scores compared to the control group	2b
Stergiou et al. [45]	Significant improvements in GMFM, GMPM, and PBS scores post-intervention, with benefits persisting 2 months after the intervention. Spasticity showed an improving trend but was not statistically significant	14 CP children with spastic quadriplegia Male = 11 Female = 3	Significant improvements in Gross Motor Function Measure, Gross Motor Performance Measure, and Pediatric Balance Scale scores post-intervention, with benefits persisting 2 months after the intervention. Although spasticity measured using Modified Ashworth Scale showed an improving trend, it did not reach statistical significance	2b

The assessment of the included papers based on the OCEBM scoring system revealed the following distribution of scores: five papers obtained a score of 4, one paper obtained a score of 3b, twenty papers obtained a score of 2b, one paper obtained a score of 1b, two papers obtained a score of 2a, and one paper obtained a score of 1.

### Methods for muscle spasticity assessment

Identified methods to scale muscle spasticity included clinical qualitative and instrumented quantitative techniques. Quantitative approaches are further classified into neurophysiological response and biomechanical response methods. The methods identified from the selected articles are explained in the following section.

#### Hofmann’s reflex

The Hoffman's reflex, commonly known as the H-reflex, is a neurophysiological technique widely used to assess muscle spasticity. This method involves stimulating a mixed peripheral nerve—usually the nerve that serves the muscles being tested—with a mild electrical current. The H-reflex is similar to the natural reflex that occurs when a muscle tendon is tapped (like in a knee-jerk reflex), but it is elicited in a controlled manner using electrical stimulation [46].

When the nerve is stimulated, it causes a response in the muscle, which is then recorded. The key aspect of the H-reflex is the measurement of the reaction time (latency) and the size (amplitude) of the muscle response. Typically, in spastic muscles, as seen in conditions like CP, the reflex response is exaggerated—meaning the muscles respond more quickly and with greater force than normal [46].

Additionally, the ratio of the maximum reflex response to the maximum direct muscle response (known as Hmax/Mmax ratio) is calculated. This ratio provides valuable information about the excitability of the spinal motor neurons controlling the muscle. However, it's important to note that there is an overlap in the values of this scale between healthy and spastic muscles, which can sometimes limit its diagnostic efficiency [46]. Furthermore, obtaining the maximum direct muscle response, which is essential for the Hmax/Mmax calculation, requires a strong stimulus that can be uncomfortable, making it less frequently used in children [46].

#### Modified Ashworth Scale (MAS)

The MAS is a clinical assessment tool used to evaluate spasticity in patients with neurological conditions such as CP, stroke, or spinal cord injury. The MAS measures the resistance of a muscle group to passive stretching on a six-point ordinal scale, ranging from 0 (no increase in muscle tone) to 4 (rigidity). A score of 1 indicates a mild increase in muscle tone with a catch and release, while a score of 2 represents a more marked increase in muscle tone through the entire range of motion, but the limb can still be easily moved. A score of 3 indicates a considerable increase in muscle tone; passive movement is complex, and there is a “catch” at a certain point in the range of motion. A score of 4 represents rigid flexion or extension [47].

The MAS is a common method of muscle spasticity assessment since it does not require any equipment and can be performed rapidly, efficiently, and in a daycare clinic [48]. The test is performed manually to assess the muscle resistance to passive stretching and was primarily defined as a scale of spasticity [48]. However, its result depends on the speed at which the test is done [48]. This drawback limits the reliability of this test and increases the chances of error in assessments. Therefore, the results of spasticity evaluations obtained with this scale should be interpreted cautiously. The other limitation of the MAS is that it only provides a subjective assessment of spasticity based on the clinician’s interpretation of the resistance to passive stretching [21]. Additionally, the MAS does not provide information on the underlying neural mechanisms of spasticity, such as changes in muscle fibre properties or altered reflex pathways [21].

#### Tardieu Scale (TS)

This test evaluates muscle resistance to both slow and fast passive motions [48]. TS assessment is simple and relatively easy to carry out. Moreover, the results of this assessment, i.e., the spasticity angle X and the spasticity grade Y, can be correlated with gait analysis if needed [22].

The Tardieu scale has excellent intra- and interrater reliability when measured at the elbow and ankle joints of children with CP. Moreover, no difference was noted between visual and goniometric assessments. The Tardieu Scale is commonly used during the evaluation of children with CP; nevertheless, it is associated with several drawbacks, such as lack of standardization, precise control over stimulation, and poor reliability and validity for qualitative and subjective assessments of all muscle groups [48].

#### Electromyography (EMG)

Electromyography (EMG) is a technique used to measure the electrical activity of muscles. It involves the placement of surface or fine wire electrodes on the skin overlying the muscle of interest, which then records the electrical activity generated by the muscle during movement [23]. EMG determines muscle activation patterns, timing and coordination, and muscle recruitment during functional tasks. In children with CP, EMG can be used to assess muscle spasticity by measuring the level of muscle activity during passive or active movement [49].

One limitation of EMG is that it only measures muscle activity on the surface, so it may not accurately reflect deep muscle activity [50]. Additionally, EMG cannot distinguish between spasticity and other factors contributing to increased muscle activity, such as compensation strategies or pain [51].

#### Pendulum test

The pendulum test, also known as the Wartenberg test, is a biomechanical method that measures muscle tone by using gravity to stimulate the muscle stretch reflex during passive swinging of the lower leg. Studies that used this method to assess spasticity in children with CP report that it may provide an objective assessment distinguishing various degrees of spasticity in this population [19, 24]. However, it needs to be clarified whether the outcomes of the pendulum test correlate with the results of other spasticity assessment methods in children with CP [19]. The pendulum test is simple, quick, and noninvasive, with reproducible results. Furthermore, it is nonintimidating to children or people with cognitive deterioration. However, its main drawback is that the test outcomes are thoroughly influenced by the level of muscle relaxation and sitting position [24].

#### Acoustic radiation force impulse (ARFI) elastography

ARFI elastography is a recently developed technique that overlays tissue elasticity data on standard images obtained with commercial ultrasound scanners. ARFI elastography systems either show a map displaying spatial differences in tissue spasticity or report tissue elasticity quantitatively as shear wave velocity (SWV), normally measured in meters per second (m/s) [52].

ARFI elastography-based quantification of tissue spasticity is a noninvasive, inexpensive, safe, and quick imaging tool with reliable and reproducible results that can improve the precision of ultrasound tests in determining muscle spasticity [52]. However, ARFI elastography is an operator skill-dependent technique that requires precision mechanical equipment, which is not easily applicable at the bedside [53].

#### Real-time sonoelastography (RTS)

Real-time sonoelastography (RTS) is another novel ultrasound-based technique that assesses the elasticity of the tissue in real time. RTS is based on the principle that tissue strain (displacement) is lower in hard tissue and higher in soft tissue [25]. However, RTS involves tissue compression, leading to imprecise outcomes and limiting interoperator reproducibility. Therefore, it can be considered a semiquantitative assessment [26].

#### Dynamic evaluation of range of movement (DAROM)

The DAROM evaluation method considers muscle stiffness, movement velocity, and adjacent joint positions to assess spasticity. The DAROM, a simplified form of the modified Tardieu Scale, demonstrated good intra- and interrater reliability when passive muscle stretching was repeated at two different speeds. The range of movement in this test is defined as slow and quick passive stretching to assess a dynamic component of muscle spasticity. Unlike standard clinical examinations, the DAROM represents a “range of movement deficit” (DROM), a value from the minimal muscle stretch position. In this test, two joint angles are measured: DROM I, described as the passive range of movement (PROM) deficiency following a slow velocity stretch, and DROM II, defined as the angle of catch after a quick velocity stretch. The difference between DROM II and DROM I demonstrates the examined muscle group’s level of spasticity and is called the angle of spasticity (AOS) [27]. The DAROM examination is a simultaneous accelerometric assessment of the range of motion ROM deficiency and the corresponding passive motion angular velocity, which enables the observer to assess the static contractures and dynamic spastic components. However, its drawback is that this measure is not an objective test [27].

#### Australian spasticity assessment scale (ASAS)

ASAS is a recently developed method to assess muscle spasticity [34]. The ASAS determines the presence of spasticity by identifying a velocity-dependent increased response to rapid passive movement. An ordinal scale is used to quantify this method. No instrument is required to perform this tool, and it is easy to apply in the clinical setting [34]. Although Sarah Love and her colleagues demonstrated promising reliability between raters, further research needs to be conducted to clarify the responsiveness of the ASAS to detect change after specific spasticity interventions [34].

#### Ely test

The Ely test (Duncan-Ely) is a clinical technique for evaluating rectus femoris spasticity [35]. It is a velocity-dependent test measured as positive or negative by quickly flexing the knee while lying prone in a relaxed state [35]. The Root-Ely test, a modified version of the Duncan-Ely test, is a 5-point numerical rating system that determines where the catch happens in the quick arc of knee flexion [35].

There are some limitations associated with this study including the lack of standardized velocity in measuring spasticity which potentially affects the consistency and accuracy of measurements [35]. Furthermore, differences in how each clinician performs the measurements may lead to inconsistencies and biases in the results [35]. Lastly, the possibility of a learned effect by the children, whereby repeated measurements influence their responses, is a challenge in reliability studies and may confound the results, and we cannot control this effect [35].

#### Hypertonia assessment tool

The hypertonia assessment tool has seven components: items one, two, and six assess dystonia, items three and four measure spasticity, and items five and seven examine rigidity [36]. The items are graded as either positive or negative [36]. One or more positive scores on one hypertonia item indicate the presence of this subtype [36]. Each limb is examined and given an individual diagnosis of hypertonia [36].

Since this method is a subjective test, to improve the quality of the results, the test procedure (e.g., hand positioning) needs to be standardized and assessors should be trained properly [36].

#### Numeric rating scale

The 0–10 numeric rating scale (NRS) is utilized to assess spasticity [37]. NRS is a self-reported outcome measure in which 0 represents no spasticity and 10 denotes the greatest spasticity [37]. This rating instrument is commonly used in clinical settings to promptly evaluate pain [37].

There are various perspectives on spasticity among children, caretakers, and clinicians, which can lead to challenges in accurately assessing and measuring spasticity using NRS [37]. Children identify spasticity with end range of movement, caretakers with generalized hypertonia, and physicians with a velocity-dependent component of spasticity [37].

#### Inertial sensors

Inertial sensors, which are lightweight devices containing accelerometers, gyroscopes, and sometimes magnetic sensors, are employed to track the movement of both proximal and distal body segments during rapid passive muscle stretch [40]. These sensors can offer insights into the angle of catch, a significant aspect of spasticity evaluation [41]. Similar to dynamometry, mathematical models have been proposed to create an objective measure of spasticity, utilizing data gathered from these sensors [13].

#### Stiffness tool (L-STIFF)

Driven Gait Orthosis Lokomat is a device created for robotic-assisted gait rehabilitation that allows patients with neurological movement disorders to simply measure the mechanical stiffness of a joint while performing robotic-assisted gait training with partial body weight support [38]. The L-STIFF tool detects changes in resistive torque in hip and knee joints during predetermined passive motions in both flexion and extension, moving the joint at a constant velocity with a regulated range of motion [38, 42].

The L-STIFF assessment technique is a viable option for automated stiffness testing in children with CP, but it is not sensitive enough to detect minor variations in muscle tone [38].

#### Tonic stretch reflex threshold

The Tonic Stretch Reflex Threshold (TSRT) is determined by stretching the spastic muscle at various fast paces while measuring the joint angle with an electrogoniometer and the myoelectric response using EMG [39]. The TSRT index is calculated using linear regression (the stretch reflex threshold angle and velocity) [39, 43]. A TSTR angle is estimated by extending the regression line until it intersects with the velocity axis at 0 degrees per second [39, 43].

## Discussion

This article aims to review and compare the available subjective scales and objective measures for assessing muscle spasticity in children with CP. Muscle spasticity is a common motor disorder that affects individuals with CP [46]. It occurs due to damage to the part of the brain that controls muscle movement and can affect any part of the body, such as the legs, arms, and trunk [46]. Muscle spasticity significantly impacts a person’s ability to perform daily activities and quality of life; hence, measuring and monitoring the level of muscle spasticity is important [19].

Various methods are used to classify muscle spasticity and aid in its management.^44^ Current subjective and objective methods to measure the spasticity of muscles include Hofmann’s reflex or H-reflex [46], the Modified Ashworth Scale [21, 47, 48], the Tardieu scale [22, 48], electromyography (EMG) [23, 49–51] pendulum tests [19, 24], acoustic radiation force impulse (ARFI) elastography [52, 53], real-time sonoelastographs (RTS) [25, 26], the dynamic evaluation of range of motion (DAROM) scale [27], the Australian spasticity assessment Scale (ASAS) [34]. Ely Test [35], hypertonia assessment tool [36], numeric rating Scale [37], inertial sensors [40, 41], stiffness tool (L-STIFF) [38, 42], and tonic stretch reflex threshold [39, 43].

These methods can be categorized into two main groups: neurophysiological response methods and biomechanical response methods. Neurophysiological response methods include techniques such as the H-reflex and EMG. The H-reflex measures the electrical response of a muscle to low-threshold electrical stimulation, while EMG measures the electrical activity of muscles during movement [23, 46]. Both methods offer insights into muscle activation patterns and motor neuron excitability but may have limitations in distinguishing spasticity from other factors affecting muscle activity.

Biomechanical response methods include clinical assessment tools such as the MAS and the TS, as well as novel techniques such as ARFI and RTS. The MAS and TS are widely used clinical tools to assess spasticity based on resistance to passive stretching, while ARFI and RTS offer noninvasive and real-time imaging approaches to quantify tissue elasticity.

In addition to these methods include the Ely Test and Hypertonia Assessment Tool, which fall under more subjective assessments due to their reliance on clinician interpretation and patient responses. The Numeric Rating Scale, while simple and commonly used, also falls into this subjective category. Conversely, Inertial Sensors and the Stiffness Tool (L-STIFF) provide more objective biomechanical response measurements by analyzing the physical properties of muscle movements and stiffness. Similarly, the Tonic Stretch Reflex Threshold (TSRT) offers an objective approach by quantifying reflex thresholds and muscle dynamics.

Reviewing the selected articles suggests that each assessment method has advantages and limitations. Some methods, such as the MAS and TS, are commonly used in clinical practice due to their simplicity and accessibility [48]. However, they may be subject to subjective interpretation and may not fully capture the underlying neural mechanisms of spasticity [21, 48].

On the other hand, newer techniques such as ARFI elastography and RTS offer more objective and quantitative measures of tissue elasticity but may require specialized equipment and operator skills. The strengths and limitations of each method are listed in Table 3.

**Table 3:** A comparison between different methods of muscle spasticity assessment in children with CP

Assessment method	Type	Measurement	Setting	Strengths
Modified ashworth scale	Subjective	Resistance to passive stretching	Clinic or inpatient	Easy to perform, no equipment needed
	Limitations: Reliability may vary depending on test speed and subjectivity of interpretation			
Tardieu scale	Subjective	Resistance to slow and fast passive motions	Clinic or inpatient	Provides spasticity angle and grade, correlation with gait analysis possible
	Limitations: Lack of standardization and interoperator variability, may not be suitable for all muscle groups			
Electromyography	Objective	Muscle electrical activity	Clinic or research	Provides detailed muscle activity data during movement
	Limitations: Limited to surface muscles, does not distinguish spasticity from other factors like pain or compensation			
Hofmann’s reflex	Objective	Neurophysiological response	Clinic or research	Indicates alpha-motor neuron excitability
	Limitations: Overlap between healthy and spastic muscles			
Acoustic radiation force impulse elastography	Objective	Tissue elasticity	Clinic or research	Noninvasive, quick, and safe
	Limitations: Operator skill dependent, requires specialized equipment			
Real-time sonoelastography	Objective	Tissue elasticity	Clinic or research	Provides real-time tissue elasticity data
	Limitations: Limited by tissue compression during assessment; not fully objective			
Dynamic evaluation of range of motion	Objective	Range of movement deficiency	Clinic or inpatient	Simultaneous assessment of static contractures and dynamic spasticity
	Limitations: Not fully objective, requires accelerometric assessment			
Australian spasticity assessment scale	Objective	Velocity-dependent response	Clinic or research	Easy to apply, nonintimidating
	Limitations: Requires further research to establish responsiveness to interventions			
Pendulum test	Biomechanical	Muscle stretch reflex	Clinic or research	Simple, quick, and noninvasive
	Limitations: Outcomes influenced by muscle relaxation and sitting position			
Ely test	Subjective	the catch occurs in the quick arc of knee flexion	Clinic or research	Specific and accurate focus on rectus femoris muscle
	Limitations: Variability in execution, lack of standardized measurement velocity			
Hypertonia assessment tool	Subjective	Spasticity, dystonia, and rigidity	Clinic or inpatient	Comprehensive assessment of hypertonia subtypes
	Limitations: Requires standardized procedure and trained assessors			
Numeric rating scale	Subjective	General spasticity perception	Clinic or inpatient	Easy and quick to use
	Limitations: Subject to individual perception and understanding of spasticity			
Inertial sensors	Objective	Movement and spasticity angle	Clinic or inpatient	Provides detailed data on movement
	Limitations: Relies on mathematical models for spasticity measurement			
Stiffness tool (L-STIFF)	Objective	Joint mechanical stiffness	Clinic or research (with Lokomat device)	Automated, precise assessment
	Limitations: May not detect minor muscle tone variations			
Tonic stretch reflex threshold	Objective	Reflex threshold and muscle dynamics	Clinic or research	Detailed analysis of reflex threshold
	Limitations: Requires specific equipment and expertise			

The review also highlights the need for more research to establish the reliability, validity, and responsiveness of newer methods such as the ASAS. Additionally, further studies could explore the correlations between the outcomes of different assessment methods to determine their complementary roles in evaluating muscle spasticity.

Incorporating multiple assessment techniques is essential for a comprehensive understanding of muscle spasticity [54]. While methods like the modified Ashworth Scale (MAS) are easily applied, they can be enhanced with objective tools like electromyography (EMG) or acoustic radiation force impulse (ARFI) elastography. This fusion enhances assessment accuracy, shedding light on neural and biomechanical factors influencing spasticity [54].

Developing standardized protocols and establishing normative data for these techniques is vital. Such standardization facilitates clinical translation of the methods and ensures consistent results across studies [55]. Additionally, longitudinal studies that track changes in muscle spasticity over time and in response to various interventions are essential to establish the responsiveness and reliability of these methods in a clinical context.

Furthermore, considering factors such as age, CP severity, and comorbidities when selecting and interpreting assessment methods is essential. Customizing evaluations to each child’s specific needs can lead to personalized treatment plans and enhanced outcomes.

The limitations of the present article review are related to the limited longitudinal data and heterogeneity of the included studies. The included studies in this review used different populations, assessment protocols, and outcome measures, leading to heterogeneity in the data. This diversity in methodologies may limit direct comparisons and the generalizability of the findings. The review mainly relies on cross-sectional studies, which may not provide comprehensive insights into the effectiveness of different assessment methods over time.

## Conclusions

Muscle spasticity assessment in children with CP is essential for an effective treatment/spasticity management plan and follow-up. Current spasticity assessment techniques are primarily subjective and lack sufficient reliability to quantify the level of muscle spasticity in children with CP. New methods that can objectively, accurately and reliably scale muscle spasticity can provide insight into each child’s condition with CP and aid physicians in optimizing personalized treatment plans. Moreover, they can assist in monitoring the efficiency of treatments.

### Supplementary Information

Supplementary Material 1.

## Data Availability

The datasets used and/or analyzed during the current study are available from the corresponding author upon reasonable request.
