# Supplementary material for: Methods of muscle spasticity assessment in children with cerebral palsy: a scoping review
Source: J Orthop Surg Res. 2024 Jul 11;19:401. doi: 10.1186/s13018-024-04894-7 (PMC11238363; doi:10.1186/s13018-024-04894-7)
Supplement: Supplementary file 1 — Supplementary Material 1. [file 13018_2024_4894_MOESM1_ESM.docx]

**Databases**: PubMed, Web of Science Core Collection, Google Scholar

**Date Range**: - to April 2024

**Keywords and Search Terms**:

1. "muscle spasticity" OR "spasticity"
2. "Cerebral Palsy" OR "CP"
3. "Children" OR "pediatric"
4. "assessment methods" OR "evaluation methods" OR "measurement tools"
5. "objective measures" OR "subjective measures"

**Search String(for all databases):**

("muscle spasticity" OR "spasticity") AND ("cerebral palsy" OR "CP") AND ("children" OR "pediatric") AND ("assessment methods" OR "evaluation methods" OR "measurement tools") AND ("objective measures" OR "subjective measures")

**Selection Criteria**:

- Articles involving children and adolescents (0-18 years of age) with cerebral palsy
- Assessments of muscle spasticity using either objective or subjective measures
- Comparisons of different assessment methods or evaluations of the effectiveness of a particular assessment method
- Studies published in English

**Exclusion Criteria**:

- Articles not involving children or adolescents with cerebral palsy
- Studies not focusing on muscle spasticity assessment
- Articles not available in English

**Procedure**:

- Initial screening of titles and abstracts
- Full-text review for eligible studies
- Duplication removal using Covidence
- Inclusion of additional articles identified through reference list reviews
- Consultation with subject matter experts for potential missing articles

Top of Form

Bottom of Form
